# Visualization of genetic disease-phenotype similarities by multiple maps t-SNE with Laplacian regularization

**DOI:** 10.1186/1755-8794-7-S2-S1

**Published:** 2014-10-22

**Authors:** Weiwei Xu, Xingpeng Jiang, Xiaohua Hu, Guangrong Li

**Affiliations:** 1 International School of software, Wuhan University, Wuhan, Hubei, 430079, China; 2 College of Computing & Informatics, Drexel University, Philadelphia, PA 19104, USA; 3 School of Business, Hunan University, Changsha, Hunan, 410012, China

## Abstract

**Background:**

From a phenotypic standpoint, certain types of diseases may prove to be difficult to accurately diagnose, due to specific combinations of confounding symptoms. Referred to as phenotypic overlap, these sets of disease-related symptoms suggest shared pathophysiological mechanisms. Few attempts have been made to visualize the phenotypic relationships between different human diseases from a machine learning perspective. The proposed research, it is anticipated, will visually assist researchers in quickly disambiguating symptoms which can confound the timely and accurate diagnosis of a disease.

**Methods:**

Our method is primarily based on multiple maps t-SNE (mm-tSNE), which is a probabilistic method for visualizing data points in multiple low dimensional spaces. We improved mm-tSNE by adding a Laplacian regularization term and subsequently provide an algorithm for optimizing the new objective function. The advantage of Laplacian regularization is that it adopts clustering structures of variables and provides more sparsity to the estimated parameters.

**Results:**

In order to further assess our modified mm-tSNE algorithm from a comparative standpoint, we reexamined two social network datasets used by the previous authors. Subsequently, we apply our method on phenotype dataset. In all these cases, our proposed method demonstrated better performance than the original version of mm-tSNE, as measured by the neighbourhood preservation ratio.

**Conclusions:**

Phenotype grouping reflects the nature of human disease genetics. Thus, phenotype visualization may be complementary to investigate candidate genes for diseases as well as functional relations between genes and proteins. These relationships can be modelled by the modified mm-tSNE method. The modified mm-tSNE can be applied directly in other domain including social and biological datasets.

## Background

A large number of studies proved that mutations of functionally related genes are associated with genetic diseases characterized with overlapping phenotypes [1,2]. On the other hand, diseases with different clinical features and genes may also have similar pathophysiological mechanisms [3,4]. Based on these assumptions, a number of studies focus on developing computational frameworks for discovering disease-related gene candidates by exploiting complex associations between phenotypes and genotypes found within heterogeneous genomic datasets such as gene expression data, protein-protein interaction networks [5,6] and gene ontology annotations [7]. Studying the associations between diseases not only help us to find their common genetic basis [8], but also provide novel insights into molecular mechanisms [9] and future drug targets for pharmaceutical research [10].

**Figure 1:**
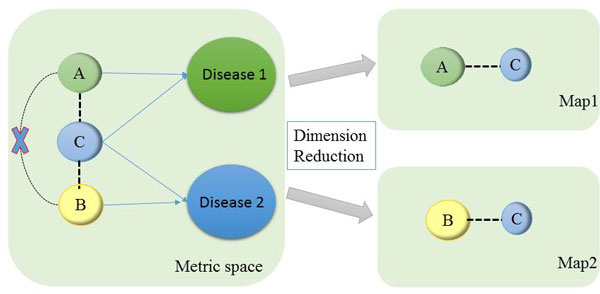
**Explanation of non-metric similarity**. The example illustrates how mm-tSNE can visualize phenotype overlapping in different maps that do not obey the triangle inequality. The size of the points corresponds to importance weights of points in each map.

It is beneficial to gain an intuitive understanding of a large dataset by first exploring and visualizing it before any computational intensive tasks are performed. For visualizing disease phenotypes, we may obtain novel insights into disease and gene relationships. Traditional techniques for visualization methods convert high-dimensional data into two or three dimensional metric spaces [11], and construct one single map for visualizing objects. The main limitation of utilizing a metric space approach is the transitivity of similarities induced by triangle inequality. For instance, if phenotype A is close to phenotype C within the metric space, and phenotype B is close to phenotype C, then logically, phenotype A must be close to phenotype B. However, in reality A may not necessarily be similar to B. Since the diseases might be related with each other in different categories, they may have overlapping phenotypes, of which the set of phenotypes may belong to different disease categories. In this paper, we take into account the nature of disease-related phenotypic data and investigate a novel method based on mm-tSNE [12] to construct several maps that visualize the non-metric similarities among phenotypes. mm-tSNE can appropriately model intransitive similarities by giving each point an importance weight in different maps. For instance, we embed three example phenotypes A, B, C into two maps in lower dimensional spaces (See Figure 1 for illustration), mm-tSNE assigns phenotype A an importance weight of 1 on the first map, phenotype B an importance weight of 1 in the second map, and phenotype C an importance weight 0.5 in both. The pairwise similarity between phenotype A and B, therefore, is 0. We employ mm-tSNE to overcome transitive similarities which break the non-metric similarity of data points to different maps [12]. The result shows that the probabilistic nature of mm-tSNE can successfully visualize non-metric phenotypes similarity.

However, mm-tSNE may have some disadvantages that high importance weight points in the same map do not correspond to the same cluster. That provide difficulty to explain the meaning of each map. We introduced a Laplacian regularization procedure for mm-tSNE. The Laplacian regularization has been used for many other objective functions such as linear regression [13] and Gaussian Mixture Model [14]. The advantage of regularization for mm-tSNE is that it adopts clustering structures of variables and provides more sparsity to estimated parameters. Our experimental results indicate that the novel method can achieve comparable performance and provide a more flexible framework for data visualization than mm-tSNE.

## Methods

### Dataset

The input phenotype similarity matrix A is a symmetric matrix in which each row (and column) corresponds to a phenotype. Phenotype similarity was constructed by van Driels et al. [15] using the Online Mendelian Inheritance in Man (OMIM) database [16,17]. The disease classification is obtained from the Human Disease Network [8], which uses plain-text to summarize the specific features of the disease. We obtained a similarity matrix P among 1,014 phenotypes within 21 disease classes. Similarities which did not exceed a threshold of 0.5 were filtered from the results.

### t-Stochastic Neighbourhood Embedding (t-SNE)

t-Stochastic Neighbourhood Embedding (t-SNE) is a method that tries to find a non-linear mapping between high dimensional space and low dimensional space that keeps distances between pairs of points, while preserving both local and global information [18]. It has been successfully applied to visualize documents [19], breast cancer CADx imaging data [20], and many other domains. t-SNE models the similarity among data points by probability distance rather than Euclidean distance. The similarity between two points in high dimensional space is represented by joint probabilistic distance$p_{ij}$:

(1) $$p_{ij}=\frac{exp\left(-||x_{i}-x_{j}|{|}^{2}\;/)\;2\sigma^{2}\right)}{\sum_{k}\sum_{l\neq k}exp\left(-||x_{k}-x_{l}|{|}^{2}\;/)\;2\sigma^{2}\right)},\;\text{for }\forall \text{i},\text{j}:\text{i}\neq \text{j}$$

The goal of t-SNE is to compute a 2 or 3 dimensional space where the probabilistic distances among all data points are preserved. t-SNE uses heavy-tailed distribution ${\text{Q}}_{\text{ij}}$ that centers at each point to define the 2 or 3 dimensional "phenotype space", in order to avoid the "crowding problem" [18]. The similarity between two points in low dimensional space is represented by joint probabilistic distance${\text{q}}_{\text{ij}}$:

(2) $$q_{ij}=\frac{{\left(1+||y_{i}-y_{j}|{|}^{2}\right)}^{-1}}{\sum_{k\neq l}{\left(1+||y_{k}-y_{l}|{|}^{2}\right)}^{-1}},\;\text{for}\;\forall \text{i},\text{j}:\text{i}\neq \text{j}$$

In t-SNE, how faithful that ${\text{q}}_{\text{ij}}$ models ${\text{p}}_{\text{ij}}$ is measured by Kullback-Leibler divergence. The cost function of t-SNE is given by:

(3) $$C=KL\left(P_{i}|Q_{i}\right)=\sum_{i}\sum_{j}p_{ij}log\frac{p_{ij}}{q_{ij}}$$

### Multiple maps t-SNE

mm-tSNE is an extension of t-SNE, which constructs several maps M to visualize non-metric properties of phenotype similarities that alleviates the limitation of one single metric map. According to the nature of input similarity matrix P in high dimensional space, we normalized the original similarity matrix A to make sure that the input similarity matrix P could be a symmetric, non-negative and sums up to one.

Phenotype similarities in two dimensional spaces are also presented by ${\text{q}}_{\text{ij}}$, which is the similarity between phenotype i and phenotype j in the visualization as the weighted sum of the pairwise similarities between the points corresponding to the input objects i and j across all M maps. The similarity matrix among all points in maps m can be written as:

(4) $$q_{ij}=\frac{\sum_{m}\pi_{i}^{\left(m\right)}\pi_{j}^{\left(m\right)}{\left(1+||y_{i}^{\left(m\right)}-y_{j}^{\left(m\right)}|{|}^{2}\right)}^{-1}}{\sum_{m^{\prime }}\sum_{k\neq l}\pi_{k}^{\left(m^{\prime }\right)}\pi_{l}^{\left(m^{\prime }\right)}{\left(1+||y_{k}^{\left(m^{\prime }\right)}-y_{l}^{\left(m^{\prime }\right)}|{|}^{2}\right)}^{-1}}\;\text{for}\;\forall \text{i},\text{j}:\text{i}\neq \text{j}$$

where ${\text{y}}_{\text{i}}^{\left(\text{m}\right)}$ represents the low-dimensional model of object i in map m. In each map m, a phenotype point i has an weight $\pi_{\text{i}}^{\left(\text{m}\right)}$, $\pi_{\text{i}}^{\left(\text{m}\right)}\geq 0$ that measures the importance of point i in map m, the weight of phenotype point i over all maps M are equal to 1.For computational convenience, the weight $\pi_{\text{i}}^{\left(\text{m}\right)}$ was represented by unconstrained weight ${\text{w}}_{\text{i}}^{\left(\text{m}\right)}$:

(5) $$\pi_{i}^{\left(m\right)}=\frac{e^{-w_{i}^{\left(m\right)}}}{\sum_{m^{\prime }}e^{-w_{i}^{\left(m^{\prime }\right)}}}$$

The cost function is the same as Eq. (3), but the optimization of the cost fuction with respect to the locations of the points ${\text{y}}_{\text{i}}^{\left(\text{m}\right)}$ in all phenotype maps and with respect to the weights ${\text{w}}_{\text{i}}^{\left(\text{m}\right)}$.

### Multiple maps t-SNE with Laplacian regularization

We improved mm-tSNE by adding a Laplacian regularization term to the cost function C(Y):

(6) $$C\left(Y\right)=KL\left(P||Q\right)=\left(1-\lambda \right)\underset{i}{\sum }\underset{j\neq i}{\sum }p_{ij}log\frac{p_{ij}}{q_{ij}}+\lambda \pi^{T}L\pi$$

where $\text{L}=\left(\text{diag}\left(\sum_{i}p_{ij}\right)-{\text{P}}_{\text{ij}}\right)$. The gradient of the regularized mm-tSNE with respect to the low dimensional map point ${\text{y}}_{\text{i}}^{\left(\text{m}\right)}$is given by:

(7) $$\frac{\partial C\left(Y\right)}{\partial y_{i}^{\left(m\right)}}=4\left(1-\lambda \right)\sum_{j}\frac{\partial C\left(Y\right)}{\partial d_{ij}^{\left(m\right)}}\left(y_{i}^{\left(m\right)}-y_{j}^{\left(m\right)}\right)$$

where ${\text{d}}_{\text{ij}}^{\left(\text{m}\right)}=||{\text{y}}_{\text{i}}^{\left(\text{m}\right)}-{\text{y}}_{\text{j}}^{\left(\text{m}\right)}|{|}^{2}$.

The gradient of the regularized mm-tSNE with respect to the weight $\pi_{\text{i}}^{\left(\text{m}\right)}$is given by:

(8) $$\frac{\partial C\left(Y\right)}{\partial \pi_{i}^{\left(m\right)}}=\sum_{j}\left(\frac{2}{q_{ij}Z}\left(p_{ij}-q_{ij}\right)\right)\pi_{j}^{\left(m\right)}{\left(1+d_{ij}^{\left(m\right)}\right)}^{-1}+\lambda L\pi$$

where $\text{Z}=\sum_{k}\sum_{\text{l}\neq \text{k}}\sum_{{\text{m}}^{\text{'}}}\pi_{\text{i}}^{{\text{m}}^{\text{'}}}\pi_{\text{k}}^{{\text{m}}^{\text{'}}}\left(1+{\text{d}}_{\text{kl}}^{{\text{m}}^{\text{'}}}\right)$.

### Neighborhood preservation ratio

Neighbourhood preservation ratio (NPR) has previously been proposed by van der Maaten, Laurens [12] as a proper measurement that how well the similarities are modelled by multiple maps. NPR measures to what extent these similarities in original space are correctly preserved in multiple low dimensional spaces. For each phenotype i, we find its k neighbourhoods (namely,${\text{N}}^{\text{i}1}$) in the original space by selecting the k highest ${\text{p}}_{\text{ij}}$-values, and find its k neighbourhood (namely, ${\text{N}}^{\text{i}2}$) in multiple maps from mm-tSNE by selecting the k highest ${\text{q}}_{\text{ij}}$-values. The NPR is defined as the average ratio of preserved neighbour numbers:

(9) $$NPR=\frac{1}{n}\sum_{i=1}^{n}\frac{\left|N^{i1}\cap N^{i2}\right|}{k}$$

where $\left|{\text{N}}^{\text{i}1}\cap {\text{N}}^{\text{i}2}\right|$ count the number of elements in a set and n is the total number of phenotypes. In this paper, we apply the same way to assess NPR that helps us to choose the numbers of maps by combining the number of maps m and λ. We choose eleven different λ from 0 to 0.01, interval by 0.001. When λ= 0, our method equals to mm-tSNE.

## Results

### Model selection and performance comparison

**Figure 2:**
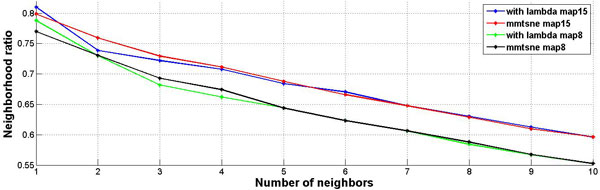
**Comparison on word association dataset**. The comparison results of neighbourhood preservation ratios for mm-tSNE and regularized mm-tSNE on word association dataset.

**Figure 3:**
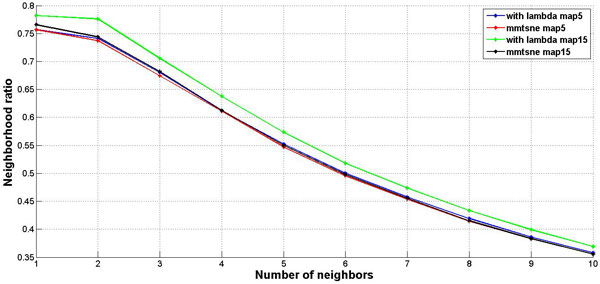
**Comparison on co-authorship dataset**. The comparison results of neighbourhood preservation ratios for mm-tSNE and regularized mm-tSNE on co-authorship dataset.

By utilizing original mm-tSNE, we have to choose one parameter m--the number of maps. But for the regularized mm-tSNE, we have an additional parameter λ. The neighbourhood preservation ratio (NPR) is applied for model selection of these parameters (See methods). Figure 2 is the comparison results on word association dataset. We compared our method with multiple maps on two datasets as previous author applied [12]. The regularized mm-tSNE has comparable performance with mm-tSNE. However, we should remind that the original version of mm-tSNE select m=8 as their best models and we selected λ=0.005, and 15 maps which reveals the relatively better neighborhood preservation ratio than other numbers. Figure 3 is the comparison results on co-authorship dataset from [12]. For this dataset, the combination of parameters λ=0.005 and m=15 reveales better neighborhood preservation ratio than other parameters. The green line in Figure 3 is our model which superior to mm-tSNE. Overall, the Laplacian regularization achieves comparable or better performance than mm-tSNE.

### Laplacian regularized mm-tSNE reveals intransitive similarity

**Figure 4:**
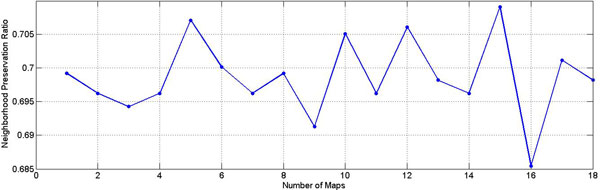
**NPR for multiple maps t-SNE on phenotype dataset**. The result shows the NPRs(neighbourhood preservation ratios) with respect to the increasing number of maps by mm-tSNE

**Figure 5:**
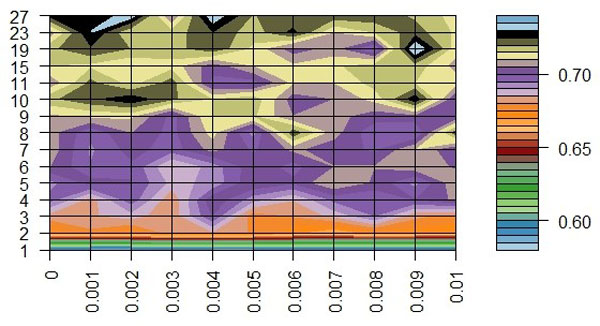
**Heatmap of neighbourhood preservation ratio for regularized mm-tSNE**. Neighbourhood preservation ratio for regularized mm-tSNE. The × axis is the values of λ we select for our experiment, y axis stands for numbers of maps. The colours of legend stand for the ratios progressively decreasing from high to low. Since the best ratio appears at λ=0.009, number of maps is 23, according to our results, we found that approximately 10 maps, λ=0.002 appear to suffice for modelling nonmetric structure of phenotype similarities.

We then applied Laplacian regularized mm-tSNE on the similarity matrix of human phenotype data for exploring the relationships among genetic-disease phenotypes. NPR with respect to number of maps is showed on Figure 4 we get highest ratio (0.708) when a 15 maps mm-tSNE is applied. Figure 5 is the heatmap of NPR in the parameter space of λ and m. The x axis is the values of λ we select for our experiment, y axis stands for numbers of maps. The colours of legend stand for the ratios progressively decreasing from high to low. The best ratio appears at λ=0.009, number of maps is 23. However, according to our results, we found that approximately 10 maps, λ=0.002 (which is the second best) appear to suffice for modelling nonmetric structure of phenotype similarities. Thus for simplification, we used the later parameters setting.

Overall, we found that phenotypes belonging to the same disease class are tend to group together. However, some phenotypes in the same disease category are overlapping with other disease class. These diseases include but not limited to developmental, skeletal diseases. This is reasonable because that most developmental disease would be expected to affect multiple tissues.

**Figure 6:**
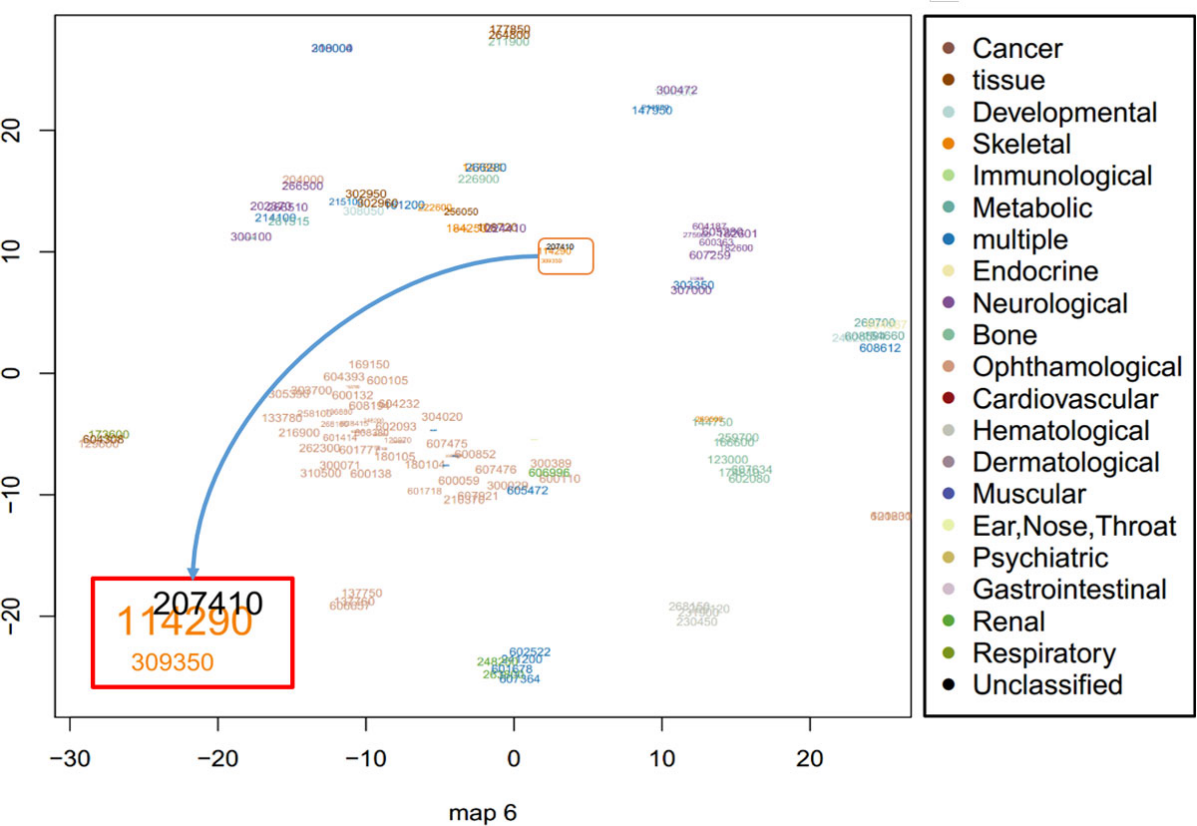
**Map 6 from regularized multiple maps t-SNE**. The results based on regularized mm-tSNE reveals one of the ten maps in which contains our selected phenotypes in examples. Each text corresponds to a specific OMIM ID. The size of each text corresponds to its importance weights in the map. The colours of each text indicated which disease categories a phenotype belongs to. We magnify the neighbourhoods details of one point Melnick-Needles syndrome (MNS, OMIM: 309350) and its two other neighbours Campomelic dysplasia (CD, OMIM ID: 114290) and Antley-Bixler syndrome (ABS, OMIM ID: 207410).

**Figure 7:**
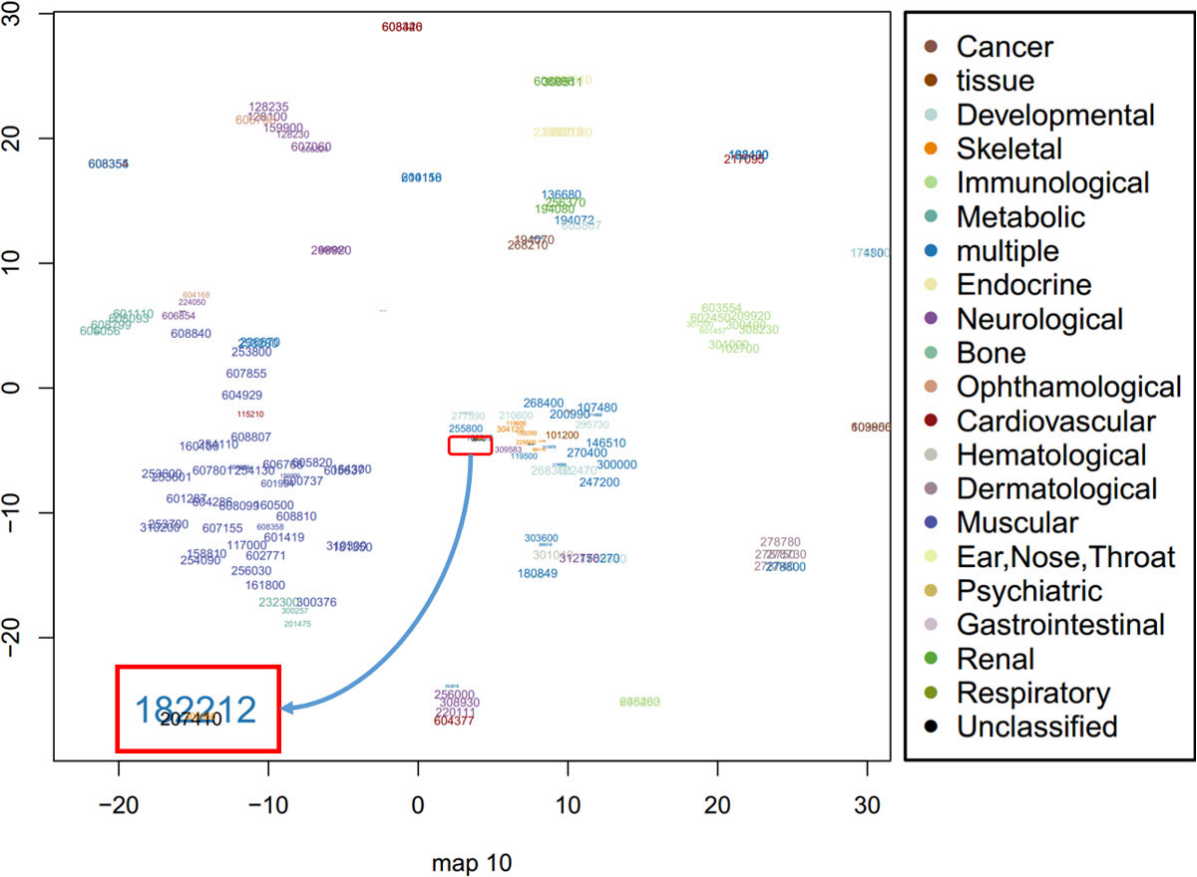
**Map 10 from regularized multiple maps t-SNE**. Based on regularized mm-tSNE, we are interested in one point--Melnick-Needles syndrome (MNS, OMIM: 309350) in map 10 as well as its neighbours Shprintzen-Goldberg syndrome (SGS, OMIM ID: 182212) and Antley-Bixler syndrome (ABS, OMIM ID: 207410). Sizes and colours of each point have the same meaning as map 6.

**Figure 8:**
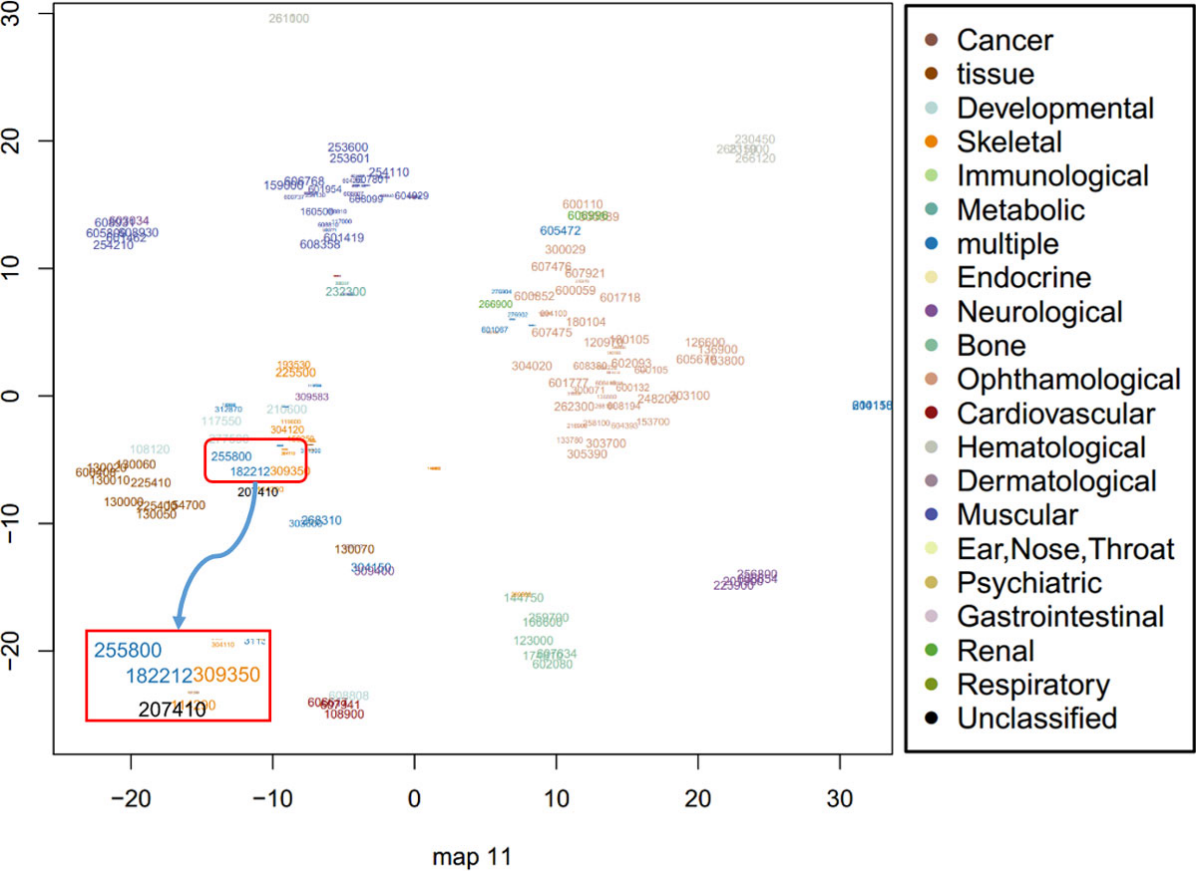
**Map 11 of 15 maps using multiple maps t-SNE**. Based on mm-tSNE, we are interested in one point--Melnick-Needles syndrome (MNS, OMIM: 309350) only in map 11. The results shows all these four phenotypes appear in one of the fifteen maps. Sizes and colours of each point have the same meaning as map 6.

Furthermore, we found that our method can appropriately model intransitive similarities between phenotypes and much better than mm-tSNE. For example, Antley-Bixler syndrome (ABS, OMIM ID: 207410) has importance weights 0.687 and 0.267 at two maps (Map 6 and 10, See Figure 6 and Figure 7) respectively. Note that to prevent the visualization from being too cluttered phenotypes with an importance weight below 0.1 were removed from each map. At Map 6, Campomelic dysplasia (CD, OMIM ID: 114290) is one of the neighbours of ABS, they have a similarity 0.502 (See Table 1) and the weights of them at metric space Map 6 are 0.687 and 0.908 respectively (See Table 2). At Map 10, ABS has a close neighbour Shprintzen-Goldberg syndrome (SGS, OMIM ID: 182212) with similarity 0.542. From Table 2 we see that SGS is not showed on Map 6 and CD is not showed on Map 10 although they are both neighbours in separate maps. That is, the neighbour of ABS in Map 6 is not necessary the neighbour of it in Map 10. Actually, the similarity of CD and SGS is 0 (See Table 1). Although the original goal of mm-tSNE is set up to discover the intransitivity of similarity, we found that mm-tSNE grouped these four phenotypes in Table 1 together in one map (See Figure 8). This result indicates that Laplacian regularized mm-tSNE reveals intransitive similarity which has not discovered by the original mm-tSNE.

**Table 1:** Extracted similarities from original matrix.

Phenotype with OMIM ID	MNS (OMIM: 309350)	CD (OMIM: 114290)	ABS (OMIM: 207410)	SGS (OMIM: 182212)
MNS (OMIM: 309350)	1	0.529	0	0.506

CD (OMIM: 114290)	0.529	1	0.502	0

ABS (OMIM: 207410)	0	0.502	1	0.542

SGS (OMIM: 182212)	0.506	0	0.542	1

Extracted similarities between four phenotypes in original similarity matrix

**Table 2:** Importance weights for extracted phenotypes.

	Map6	Map10
MNS (OMIM: 309350)	**0.460**	0.102

CD (OMIM: 114290)	**0.908**	0.073

ABS (OMIM: 207410)	**0.687**	**0.267**

SGS (OMIM: 182212)	0.002	**0.594**

The importance weights of four phenotypes in Map 6 and Map 10.

Besides CD, at Map 6 (see Figure 6) ABS has another close neighbour --Melnick-Needles syndrome (MNS, OMIM: 309350) with a similarity 0.502. ABS, CD and MNS are all neighbours at Map 6. However, it is surprisingly to see that the similarity between ABS and MNS is 0 (See Table 1). We then investigate these three phenotypes further. MNS is a skeletal disease that associated with abnormal skeletal development, as well as other health-related problems. Some main symptoms of it include short stature, abnormally long fingers and toes, irregular ribs [21]. ABS is belongs to an unclassified disease, but they share the most common symptoms [22]. CD is a severe disorder that affects the development of the skeleton and reproductive system. Although these three disorders are in three different categories (Skeletal, unclassified and developmental respectively), the common symptoms is that they are all related to skeleton system and they are often life-threatening in the new born period. The analysis shows that although the direct similarity between ABS and MNS is 0 as measured by the text mining approach from [15], our method indeed inferred their real relationships from the data. This is not inconsistent with the modelling of intransitive similarity because they are in the same metric space Map 6.

## Conclusion

We develop a novel visualization method--graph Laplacian regularized mm-tSNE. The regularization of mm-tSNE put more sparsity to the weights of data points in different maps and less sparsity to the coordinates of data points than previous method. By doing this, we got better visualization results and novel biological interpretation. On the application of this method, we found that our approach can identify interesting intransitive similarity among disease phenotypes. This approach also adds more flexibility for visualization tasks. For example, we can adjust the parameter λ to provide the weights (and the coordinate of data points in low dimensional space) more or less sparsity to "zoom in" or "zoom out" data points in different maps. We expect the new technique could be useful in more general visualization analysis in other field.

## Competing interests

The authors declare that they have no competing interests.

## Authors' contributions

WX and XJ implemented regularized mm-tSNE algorithm and run the experiments. XJ and XH designed algorithm based on mm-tSNE. GL and XH involved in the data generation and statistical analysis. All authors read and approved the final manuscript.

## Declarations

Publication of this article has been funded by the NSF IIP 1160960, NNS IIP 1332024

This article has been published as part of *BMC Medical Genomics *Volume 7 Supplement 2, 2014: IEEE International Conference on Bioinformatics and Biomedicine (BIBM 2013): Bioinformatics in Medical Genomics. The full contents of the supplement are available online at http://www.biomedcentral.com/bmcmedgenomics/supplements/7/S2.
